# Editorial: Creative Performance in Extreme Human Environments: Astronauts and Space

**DOI:** 10.3389/fpsyg.2021.709676

**Published:** 2021-06-23

**Authors:** Henderika de Vries

**Affiliations:** ^1^Yale University, New Haven, CT, United States; ^2^International Space University, Illkirch-Graffenstaden, France

**Keywords:** research agenda, creativity, extreme human environments, astronauts, space

Exploration of the universe increases the need to gain insight on creative performance in extreme environments, as astronaut's and cosmonaut's survival may even depend on it. Although the pandemic underscores its relevance, the present Frontiers Topic was initiated before the onset of this extreme human environment on Earth, stemming from the Guest Editor's work experience with gifted, and space enthusiastic students before her Ph.D. and Space studies. The development of a domain of creativity “not when it is easy”—a double entendre referring to the famous words by President JFK on going to the moon and other things, in his words “not because they are easy”—represents a contrast to the generally focused on optimal environments. The composition of the presented contributions demonstrates the newness of this research domain. From the innovative articles, a research agenda emerges, which will be discussed in the next paragraphs.

The opinion article “*The Overview Effect and Creative Performance in Extreme Human Environments”* (White), by the author of the “*Overview Effect,”* offers a rich description of this phenomenon, and includes a call to investigate scientifically the report of positive, and possibly related, effects. The author paints a colorful account of astronaut's and other explorer's experiences, and this demonstrates the contrast in space between calmly gazing out of the cupola, and at the same time the constant preparedness for potential problems, which might in his words be “two sides of the same coin,” because relaxation and meditation could enhance creative performance under stress. White further suggests focusing on research on earth-gazing in relation with the “Overview Effect.”

The brief research report “*Creating Ambassadors of Planet Earth: The Overview Effect in K12 Education”* (van Limpt-Broers et al.) represents a first empirical study on the “Overview Effect” of children (ages 9 and 10), induced by a virtual reality (VR) experience, a simulation of viewing Earth from space (“SpaceBuzz”). The simulation proved effective and as measured with self-report survey's, created awe and the “Overview Effect,” specifically through the feeling of presence. The study also demonstrated learning effects of awe for children with lower prior knowledge, and via the “Overview Effect.” The authors found an intricate gender difference of the angle of eye gaze, and a relation with the overview experience, in line with White's suggestion. The authors propose that more future research therefore could focus on learning through VR experience.

The opinion article “*Space for STEAM: New Creativity Challenge in Education”* (de Vries), proposes to integrate knowledge on scientific creative cognition within STEAM education, specifically issues related with cultural differences which impact teaching and learning. The combination of different research approaches could shine a light on how practical and cultural differences in teaching might foster or hinder development of creative cognition. An example is given, based on the “Cultural Actuation Model” (CAM) of creativity (de Vries, 2018). The CAM, which is based on empirical research, relates values of cultural contexts to the fostering of higher, or lower, original, and more, or less, fluent creative performance. The example (in the **Supplementary Material**) shows four different cultural contexts related to values of “Tolerance of Ambiguity and Uncertainty” and “Power Distance” to these different kinds of creativity.

The Hypothesis and Theory article “*The Big Bang of Originality and Effectiveness: A dynamic Creativity Framework and Its Application to Scientific Missions”* (Corazza and Lubart) presents a Space-Time Model, which represents an application of the CAM's methodology to an adult work environment (e.g., during a mission), by distinguishing as well four contextual quadrants which are related to the cultural value of “Tight-Looseness” and higher or lower original, and more, or less, fluency, of creative potential, together with the concept of intelligence. The authors embed their Space-Time model further in the theory of the Dynamic Universal Creativity Process (DUCP) which originates creativity in the “Big Bang.” The original DUCP theory therefore extends creativity principles beyond humans. The authors invite future researchers to carry out research on the “material” and “biological” layers.

The conceptual analysis “*What does Safety Mean in Safety-Critical Environments?”* (Bourgeois-Bougrine) offers an in-depth analysis of underlying mechanisms and psychological dimensions of creative processes related with insight, improvisation and creative problem-solving in life-critical situations. The author not only offers a comprehensive overview on distinct concepts, but explains their relations with creativity, and foresees implications for research in neuro-ergonomics, specifically the neural basis of creative insight problem-solving in life-critical situations. The author suggests that future research on differential psychology could concern the traditional attributes of creative individuals in safety contexts for operational performance, such as for training in simulators and other virtual reality environments.

The conceptual analysis on “*The creative Brain Under Stress': Considerations for Performance in Extreme Environments”* (Vartanian et al.) explains the altered dynamic interaction during creative performance under acute and chronic stress of the Default Network (DF), Executive Network (ECN), and the Salience Network (SN). The authors give an overview of existing knowledge on findings related to internally related thought, external sensory input, and the role of attention to maximize survival in extreme situations. In general creativity is thought to be negatively impacted by stress. The authors suggest to further scientific knowledge by continuing research on network neuroscience under stress conditions, also including during positive experiences in space as with the “Overview Effect,” to determine possible relations with creative performance.

The conceptual analysis “*How the Immune System Deploys Creativity: Why We Can Learn From Astronauts and Cosmonauts”* (de Vries and Khoury-Hanold) proposes that the immune system is a contributing factor within a multivariate theory of creativity. This entails that future research could search for individual differences in three subdomains. These concern (1) analysis of individual differences on how the immune system regulates (creative) behavior and cognition, (2) individual differences on if some people's immune system reacts more creatively than another person's immune system to an environment or aggressor (variety and number of diseases), and (3) because properties of the immune system show a surprising amount of parallels with the creative processes (e.g., divergence and convergence), future research could search for individual differences in creative properties of the immune system itself. Athletes (performing in an imagined extreme environment), as well as astronauts, are an interesting group to investigate this subject further.

The conceptual analysis on “*Creativity and Cognition in Extreme Environments: The Space Arts as a Case Study*” (Hays et al.) represents a socio-cultural perspective on this Research Topic. The article emphasizes the differences of the Earth and space environmental factors and proposes to apply the 4E cognition framework. This framework relates creative cognition to embodiment, enactment, and embeddedness in an (space or Earth) environment. Their authentic taxonomy to situate the different forms of space art distinguishes the dimensions of (1) where art is created, and (2) where art is experienced. The authors foresee that new sub-disciplines, or branches, will emerge, according to art disciplines (e.g., fashion, architecture).

The opinion article “*Exploring Similarities Across the Space and Theatre Industries”* (Chterev and Panero), compares domain-specific and domain-general characteristics between actors and astronauts. The article discusses remarkable parallels through issues such as creative problem solving, personality traits, social skills, isolation, and pretend and simulated environments. The authors suggest that the development of the field of research on creativity in extreme human environments could benefit from insight on transferable factors, also to fill gaps in literature.

Each contribution suggested new or overlapping future agenda points, which can now broadly be categorized into 5 branches of the research agenda, or in space terms, research galaxies. The first branch could be called (1) “*Classic Creativity in Extreme Environments,”* which includes all psychological contributing factors which are maybe altered in space. Within this branch there are subdomains such as virtual creativity, group creativity, and developmental issues. This further includes a variety of approaches such as socio-cultural analysis of creativity, as space offers the unique chance to study “a culture in the making.” All knowledge can be applied on Earth, for example in the educational domain. It could be argued that all existing “Earth-bound” creativity research should be replicated in an extreme environment as space because dynamics and correlations could differ with those found on Earth. However, as broad as this generalization might seem, it would limit and miss riveting possibilities of research on creativity in extreme human environments. This Research Topic overall demonstrates that the field can expand into unknown realms. Examples are a future branch of (2) “*Medicine and Creativity,”* which includes the immune-system, as well as the eye-gazing, and a third branch of (3) “*Theoretical Principles of Creativity,”* concerning creativity expanding away from human creativity, such as the DUCP. Future research in this branch might well lead to additional insights on the phenomenon of the concept itself, as well as human creativity. Another branch concerns (4) “*Experience and Creativity*,” a domain which largely exists theoretically. Studies during scientific missions are well-suited to gain knowledge on this under-researched subject. Finally, a fifth branch could be called (5) “*New Creativity,”* the appearance of new human and maybe even alien forms of creative expression in space. In sum, and continuing the use of space vocabulary, future research exploration might discover additional new galaxies of the universe of insight on creativity.

## Author Contributions

The editorial is written by HV.

## Conflict of Interest

The author declares that the research was conducted in the absence of any commercial or financial relationships that could be construed as a potential conflict of interest.
